# A Rare Case of Hemorrhagic Pericardial Effusion With Cardiac Tamponade Caused by Needle Embolism in an Intravenous Drug User

**DOI:** 10.7759/cureus.23825

**Published:** 2022-04-04

**Authors:** Swarup Sharma Rijal, Roopika Reddy

**Affiliations:** 1 Department of Internal Medicine, Tower Health Medical Group, Wyomissing, USA; 2 Department of Pulmonology and Critical Care, Tower Health Medical Group, Wyomissing, USA

**Keywords:** pericardial tamponade, needle embolism, hemorrhagic pericardial effusion, cardiac tamponade, drug user

## Abstract

Needle embolization in the heart leading to pericardial effusion with cardiac tamponade is rare. We present an unusual case of an intravenous drug user presenting with severe anginal pain with a history of intravenous needle use one month back, whose bedside echocardiography demonstrated pericardial effusion leading to tamponade. Emergent open sternotomy and exploration revealed an inadvertent tuberculin needle and hemorrhagic pericardial effusion. After removal of the needle, subsequent multiple follow-up echocardiography did not demonstrate reoccurrence of effusion.

## Introduction

Pericardial effusion occurs when there is an abnormally large amount of fluid in a sac surrounding the heart [1]. Hemorrhagic pericardial effusion is often a life-threatening medical condition mostly caused by systemic conditions such as malignancy or complications of myocardial infarction. It may develop after percutaneous cardiac interventional procedure or postpericardiotomy syndrome [2]. Needle embolism is a rare cause of pericardial effusion and a rare complication in intravenous (IV) drug abusers with the potential consequence of cardiac tamponade [3]. Needle embolism occurs when a needle fragment breaks and reaches the systemic vasculature in an IV drug user [4]. Foreign body like needle in the myocardium and pericardium is very rare with fatal outcome [4]. We hereby describe the importance of high clinical suspicion for needle embolism in the case of a young IV drug user presenting with chest pain and cardiac tamponade.

## Case presentation

A 38-year-old male smoker with a history of IV drug use presented to the emergency department with severe progressive chest pain and back pain that started abruptly one day prior to presentation with no other associated symptoms. One day prior to presentation, he was working out in the gym with vigorous resistance exercise. He had an unremarkable past medical history. He did not have a recent history of trauma, surgery, or gastrointestinal or respiratory infection. He reported last using IV heroin 36 days prior to the current presentation. He has been free of cocaine for the last three years and from amphetamine for five years.

**Figure 1:**
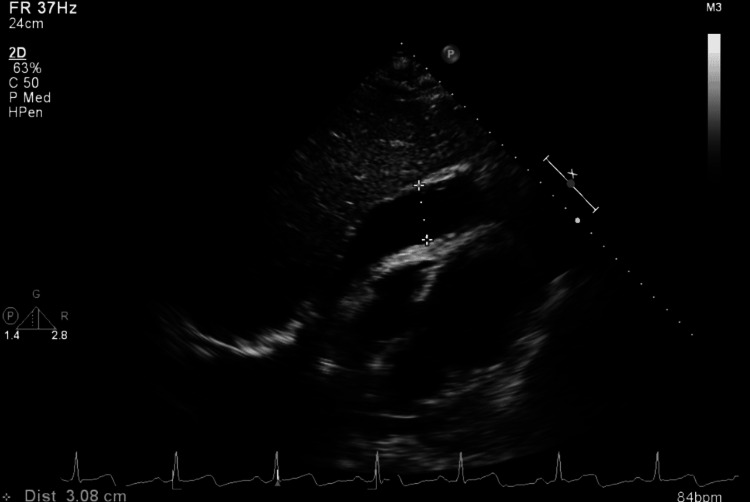
Transthoracic echocardiogram in subcostal four-chamber view demonstrating large loculated pericardial effusion.

**Figure 2:**
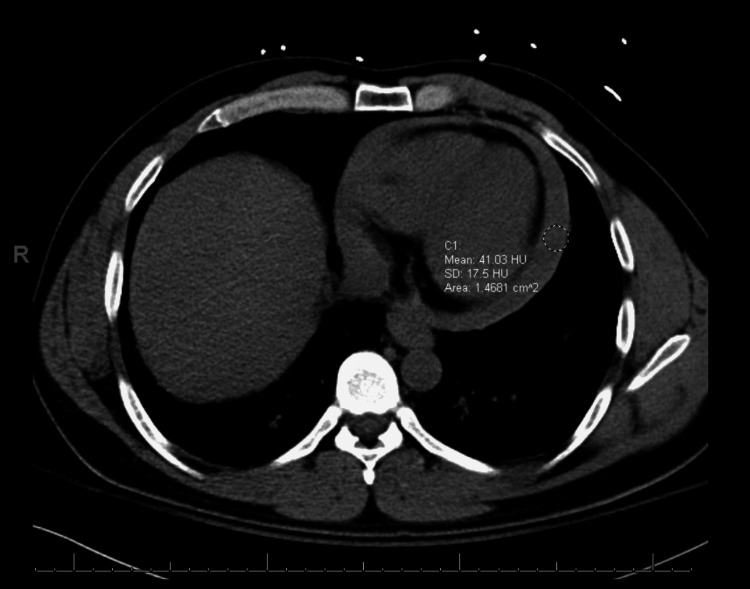
CT angiography of the chest demonstrating large hyperdense pericardial collection denoted as a circle.

Shortly after the presentation to the emergency department, he had two syncopal episodes associated with facial cyanosis, diaphoresis, and labored breathing. He was alert, and his estimated level of chest pain was 9/10. He developed mottling of his abdomen and lower extremities with rigors. On physical examination, he was afebrile and normotensive with a blood pressure of 110/60 mmHg and a heart rate of 92 beats per minute. Electrocardiography (ECG) showed normal sinus rhythm with no ST or T wave changes. With concern for acute aortic dissection, the patient was rushed for computed tomography (CT). During the scan, the patient became hemodynamically unstable with a blood pressure of 70/50 mmHg, and fluid resuscitation was initiated. A preliminary report from CT angiography did not show acute aortic dissection. Hemodynamics of the patient further worsened including blood pressure despite appropriate fluid resuscitation, and the patient required initiation of vasopressors. On auscultation, all heart sounds were muffled. Transthoracic echocardiography was performed at the bedside, which demonstrated large loculated effusion, particularly toward the inferior lateral and apical inferior portion (Figure 1). It did not have classic evidence for clear-cut aortic dissection. Systolic and diastolic functions were normal. Dilated inferior vena cava with blunted inspiratory collapse suggesting increased right-sided filling pressure was noted. The final CT angiography report revealed a large hyperdense pericardial collection (Figure 2). Because of hemodynamic instability and echocardiography findings, the patient was immediately transferred to the operating room.

The cardiothoracic surgery team performed an open sternotomy and exploration. The pericardium was noted to be very tight with no visualization of the cardiac silhouette. The pericardium was opened with the release of 500 cc of frank blood with clots and resolution of the tamponade. Careful inspection of the heart did not yield any abnormal pathology or injury. Some of the effusion had spilled into open right pleural space, and a broken hollow needle was identified. The needle was removed, and a drain was introduced into the right pleural space. The postoperative course was uneventful. A follow-up echocardiogram after two days showed grossly normal pericardium with no effusion and normal systolic function with grade 1 diastolic dysfunction. The patient was discharged on postoperative day 3 for physical rehabilitation. The patient followed up with the cardiothoracic surgery clinic three weeks after discharge with no active complaints. A limited echocardiogram at that time showed small-to-moderate pericardial effusion without tamponade physiology with normal systolic and diastolic function. However, repeat echocardiography after four months did not show pericardial effusion.

## Discussion

IV drug use is associated with multiple medical problems, and needle fragment embolization is one of them. Though appears rare, it is common as many asymptomatic needle embolization may undergo undiagnosed and drug abusers usually do not seek medical attention unless critically ill. In one retrospective study, 20 % of IV drug users among 70 mentioned a history of broken needle fragments during drug use [5]. Among the cases that have been reported so far, chest pain and dyspnea were the common presentations; however, needles could be well tolerated in many without symptoms for many years [4]. Complications of needle embolization include cardiac perforation, tamponade, infective endocarditis, and recurrent pericarditis [6]. Among the complications, to our knowledge, there are only a few cases of needle embolization causing pericardial effusion leading to cardiac tamponade [7].

Pericardial tamponade is a life-threatening condition that causes hemodynamic compromise, with the rate of fluid accumulation playing a more crucial role than the volume [8]. Bedside echocardiography is an easily available non-invasive imaging modality that helps in the rapid assessment of a patient’s clinical status and making further management plans [9]. In our case as well, fast-track bedside echocardiography was critical in therapeutic guidance.

This case also demonstrates how a small embolic needle may be missed in routine imaging such as echocardiography and CT angiography. Interestingly, in our case, the patient had a history of IV drug use one month prior to presentation and tolerated the needle for a month without any complications. However, on presentation, the patient had severe angina and was hemodynamically unstable with the tamponade phenomenon. After multidisciplinary expert discussion and with ongoing instability of the patient, open exploratory sternotomy was thought to be beneficial and the patient tolerated it well with an excellent outcome.

## Conclusions

Though rare, needle embolization to the heart should be one of the differential diagnoses in IV drug abusers presenting with chest pain irrespective of the timing of last IV drug use. As we illustrated, the patient may present serious complications with hemorrhagic pericardial effusion with obstructive shock from cardiac tamponade. A thorough history, physical examination, and early relevant imaging are crucial for diagnosis. In symptomatic patients, a multidisciplinary approach with early sternotomy and exploration of the mediastinal cavity along with the removal of the needle is key for a good outcome.
